# Effects of Fenofibrate and Gemfibrozil on Kynurenic Acid Production in Rat Kidneys In Vitro: Old Drugs, New Properties

**DOI:** 10.3390/life13112154

**Published:** 2023-11-02

**Authors:** Izabela Zakrocka, Tomasz Kocki, Ewa Urbańska, Wojciech Załuska

**Affiliations:** 1 Department of Nephrology, Medical University, Jaczewskiego Street 8, 20-954 Lublin, Poland; wojciech.zaluska@umlub.pl; 2 Department of Experimental and Clinical Pharmacology, Medical University, Jaczewskiego Street 8b, 20-090 Lublin, Poland; tomasz.kocki@umlub.pl (T.K.); ewa.urbanska@umlub.pl (E.U.)

**Keywords:** kynurenic acid, kidney, fenofibrate, gemfibrozil, hypertriglyceridemia

## Abstract

Kidney dysfunction significantly increases the cardiovascular risk, even in cases of minor functional declines. Hypertriglyceridemia is the most common lipid abnormality reported in patients with kidney disorders. PPAR-α (peroxisome proliferator-activated receptor-α) agonists called fibrates are the main agents used to lower triglyceride levels. Kynurenic acid (KYNA) is a tryptophan (Trp) derivative directly formed from L-kynurenine (L-KYN) by kynurenine aminotransferases (KATs). KYNA is classified as a uremic toxin, the level of which is correlated with kidney function impairments and lipid abnormalities. The aim of this study was to analyze the effect of the most commonly used triglyceride-lowering drugs, fenofibrate and gemfibrozil, on KYNA production and KAT activity in rat kidneys in vitro. The influence of fenofibrate and gemfibrozil on KYNA formation and KAT activity was tested in rat kidney homogenates in vitro. Fenofibrate and gemfibrozil at 100 µM–1 mM significantly inhibited KYNA synthesis in rat kidney homogenates. Both fibrates directly affected the KAT I and KAT II isoenzyme activities in a dose-dependent manner at similar concentrations. The presented results reveal the novel mechanism of action of fibrates in the kidneys and suggest their potential role in kidney function protection beyond the well-known anti-hyperlipidemic effect.

## 1. Introduction

Chronic kidney disease (CKD) remains a major health care problem, significantly increasing patient morbidity and mortality, as well as the risk of atherosclerosis and cardiovascular complications [1,2]. In CKD, an abnormal lipid profile mainly results from compromised degradation of triglyceride-rich lipoproteins [3] and contributes to increased oxidative stress and post-translational modifications of lipids [4]. Deposition of lipids in the kidney may cause direct glomerular injuries, together with mesangial cell activation and proliferation, resulting in progressive kidney damage [5]. Fibrates and peroxisome proliferator-activated receptor-α (PPAR-α) agonists are commonly used in the treatment of hypertriglyceridemia [6]. Furthermore, fenofibrate and gemfibrozil have been reported to have antioxidative properties in animal models of diabetic nephropathy [7] and aging [8].

The exogenous amino acid tryptophan (Trp) is metabolized in the body mainly through the kynurenine (KYN) pathway. The role of KYN metabolites, collectively called kynurenines, in the pathogenesis of brain diseases is well documented [9]. Increasing amounts of data also implicate kynurenines in the pathogenesis of kidney disorders, particularly of CKD. Chronic inflammation related to primary kidney disease and an impaired kidney function evoke and increase the peripheral levels of kynurenines, including KYN itself and its metabolite kynurenic acid (KYNA) [10]. Similarly, elevated activities of KYN pathway enzymes, especially kynurenine aminotransferases (KATs), have been demonstrated in CKD models [11]. As KYN is not the end-product of Trp degradation and its serum level does not clearly relate to glomerular filtration, it was proposed as a less sensitive marker of kidney function in contrast to KYNA [11]. 

In the brain and in the periphery, KYNA is synthesized by KATs, of which KAT I and KAT II are the most studied isoenzymes. KYNA acts as an antagonist of all three glutamatergic ionotropic receptors, an antagonist of α7-nicotinic acetylcholine receptors, an agonist of the G-protein-coupled receptor (GPR35) and a ligand of the aryl hydrocarbon receptor (AHR) [12,13]. AHR activation has been shown to be crucial for kidney development and normal kidney function regulation. In animal models and humans with CKD, increased AHR expression occurs in the kidneys and in extra-renal tissues, suggesting the involvement of AHR in CKD-related multiorgan damage [14]. Uremic toxins accumulating in the serum of CKD patients include KYN, KYNA, indoxyl sulfate and indole-3-acetic acid. These substances are well-described AHR ligands and are linked to a higher risk of cardiovascular events, cognitive function impairments and mortality in this group of patients [15,16,17]. 

A deficiency of KYNA, which exhibits neuroprotective properties in various experimental scenarios, was implicated in the development of neurodegeneration [18]. In the periphery, KYNA was reported to induce natriuresis [19] and to lower heart rates [20]. However, it was recently proposed as a protein-bound uremic toxin [21]. Lower KYNA clearance due to an impaired kidney function seems to be related to metabolic complications in CKD patients, especially those with higher triglycerides levels [22]. Furthermore, KYNA may induce endothelial damage, resulting in an elevated cardiovascular risk [23].

Considering that fibrates were shown to affect the kidney function [24] and that a high-fat diet elevates KYNA levels [25,26], the goal of this study was to analyze for the first time the effect of two of the most popular fibrates, fenofibrate and gemfibrozil, on KYNA formation and KAT activity in rat kidneys in vitro.

## 2. Materials and Methods

### 2.1. Animals

The experimental animals were male Wistar rats (Experimental Medicine Center, Lublin, Poland) weighing 150–200 g and aged 7 weeks, which were kept under standard laboratory conditions (12 h light–dark cycle, standard humidity and temperature 21 °C ± 1 °C), with food and water available without limits. Wistar rats are widely used in cardiovascular and metabolic research [27], mainly due to their normalized blood pressure [28]. Males were tested in our study to reduce the potential impact of hormonal variation compared to female rats. In total, kidneys from 6 animals were used in this study. All presented experiments were carried out in the morning (between 7.00 a.m. and 1.00 p.m.). All experimental methods presented in this study were in accordance with the international, national and local guidelines (Local Ethics Committee for Animal Experiments in Lublin, Poland) for the care and use of animals (based on the protocol no 32/2014).

### 2.2. Substances

The KYNA precursor, L-kynurenine (sulfate salt), the studied drugs (fenofibrate, gemfibrozil), solvent (dimethyl sulfoxide (DMSO)), the components of Krebs–Ringer buffer (sodium chloride, potassium chloride, magnesium sulfate heptahydrate, calcium chloride anhydrous, sodium phosphate dibasic dodecahydrate, sodium phosphate monobasic dihydrate, glucose), the substances essential for KAT activity analyses (Trizma base, acetic acid, pyridoxal 5′-phosphate hydrate, 2-mercaptoethanol, pyruvate and glutamine) were obtained from Sigma-Aldrich (St. Louis, MO, USA). Reagents used for high-performance liquid chromatography (HPLC) were purchased from J.T. Baker Chemicals (Phillipsburg, NJ, USA) and from Sigma-Aldrich (St. Louis, MO, USA). Detailed information about the chemical substances used in this study is available in Table 1.

### 2.3. De Novo KYNA Production in Rat Kidneys In Vitro

Rat kidneys were collected soon after animal decapitation and put on ice. Every kidney was carefully weighed and homogenized in ready-made oxygenated Krebs–Ringer buffer at pH 7.4 (1:4; *w*/*v*). Subsequently, a volume of 100 µL of kidney homogenate was transferred into test tubes previously filled with oxygenated Krebs–Ringer buffer (800 μL of buffer in every tube). Afterwards, the kidney homogenate was incubated for 2 h at 37 °C in the presence of the KYNA precursor, 10 μM L-kynurenine (50 µL volume) and one of the analyzed fibrates: fenofibrate or gemfibrozil (50 µL volume). Six different concentrations of each drug in increasing concentrations were tested: 1 μM, 10 μM, 50 μM, 100 μM, 500 μM and 1 mM. Due to the reduced water solubility, all analyzed drugs were dissolved in DMSO. For each set of experiments, six independent homogenate samples were tested (*n* = 6). Tissue incubation was terminated on ice by adding 1 N HCl (100 μL volume per each sample). At the end, all kidney samples were centrifuged (15,133× *g*, 15 min), then the obtained supernatants were analyzed by HPLC (Thermo Fisher Scientific HPLC system, ESA catecholamine HR-80, 3 μm, C18 reverse-phase column (Waltham, MA, USA)) and the KYNA concentration was evaluated fluorometrically. Each experiment was performed three times to ensure the reproducibility of results.

### 2.4. KAT I and KAT II Activity Analyses in Rat Kidneys

KAT activities in rat kidneys were analyzed as reported previously [29] with minor adjustments. Briefly, to assess KAT I and KAT II activities, animal kidneys were quickly homogenized in dialysate buffer (1:9; *w*/*v*) made from 5 mM tris-acetate buffer (pH 8.0), 50 μM pyridoxal 5′-phosphate and 10 mM 2-mercaptoethanol. Later, the obtained tissue homogenate was centrifuged (15,133× *g*, 15 min) and the collected supernatant was dialyzed with 4 L of preformed dialysate buffer for 12 h at 8 °C in ready-prepared cellulose membrane dialysis tubing. Next, the pooled enzyme sample was incubated for 2 h at 37 °C with L-kynurenine (2 µM) and the tested fibrate (in increasing concentrations: 1 μM, 10 μM, 50 μM, 100 μM, 500 μM and 1 mM) at, respectively, pH 9.5 or 7.0, which is optimal for KAT I or KAT II activity. The KAT I inhibitor glutamine (2 mM) was added to enzyme samples intended to analyze the KAT II activity. The reaction was stopped by transferring all tested samples into an ice-cold bath. At the end, the obtained samples were centrifuged and subjected to HPLC analysis, as described above. All assays were performed in triplicates. Analyses were repeated three times to obtain valid results.

### 2.5. Statistical Analysis

Experimental data were presented as means ± standard deviation (SD) unless stated otherwise. Statistical analyses were performed using a one-way analysis of variance (one-way ANOVA) followed by Tukey’s multiple comparison test (GraphPad Prism 6). Statistical significance was set at *p <* 0.05.

## 3. Results

### 3.1. Evaluation of KYNA Formation in Rat Kidneys In Vitro in the Presence of Fibrates

**Figure 1:**
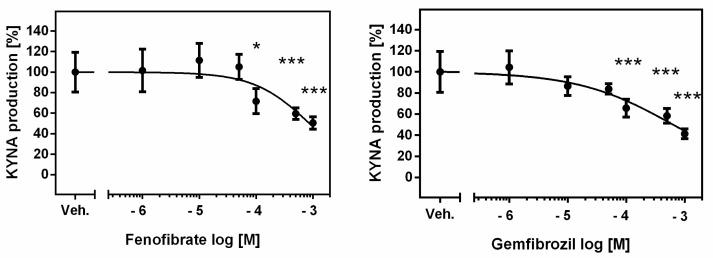
Effect of fibrates on KYNA production in rat kidneys in vitro. Data are shown as percentage of KYNA synthesis, means ± SD, *n* = 6, and are plotted against drug concentration on a logarithmic scale. ANOVA followed by Tukey’s multiple comparison test. The figure was prepared in GraphPad Prism 6. * *p <* 0.05, *** *p <* 0.001.

The de novo production of KYNA in analyzed rat kidney homogenates in the presence of 10 μM KYN was 9.95 ± 2.69 pmol/mg tissue. Fenofibrate at 100 µM, 500 µM and 1 mM concentrations suppressed KYNA synthesis to 72% (*p <* 0.05), 60% (*p <* 0.001) and 51% (*p <* 0.001) of control value, respectively (Figure 1).

Similarly, gemfibrozil at 100 µM, 500 µM and 1 mM concentrations decreased the basal KYNA production in rat kidneys in vitro to 66% (*p <* 0.001), 58% (*p <* 0.001) and 41% (*p <* 0.001), respectively (Figure 1).

### 3.2. Evaluation of KAT I Activity in Rat Kidneys In Vitro in the Presence of Fibrates

**Figure 2:**
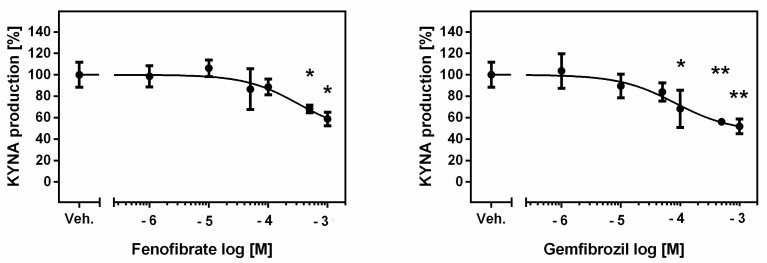
Effect of fibrates on KAT I activity in rat kidneys in vitro. Data presenting the enzymatic activity as percentage of KYNA synthesis, mean ± SD, *n* = 3, are plotted against drug concentration on a logarithmic scale. ANOVA followed by Tukey’s multiple comparison test. The figure was prepared in GraphPad Prism 6. * *p <* 0.05, ** *p <* 0.01.

The mean KYNA production by KAT I in the examined kidney homogenates in the presence of 2 μM KYN was 59.12 ± 6.98 pmol/mg tissue. Only fenofibrate at 500 µM and 1 mM concentrations inhibited KAT I activity in rat kidneys in vitro to 68% (*p <* 0.05) and 59% (*p <* 0.05) of the control value, respectively (Figure 2).

Gemfibrozil was a more potent KAT I inhibitor, suppressing this enzyme’s activity in rat kidneys in vitro at 100 µM, 500 µM and 1 mM concentrations to 68% (*p <* 0.05), 56% (*p <* 0.01) and 52% (*p <* 0.01) of the standard value, respectively (Figure 2).

### 3.3. Evaluation of KAT II Activity in Rat Kidneys In Vitro in the Presence of Fibrates

**Figure 3:**
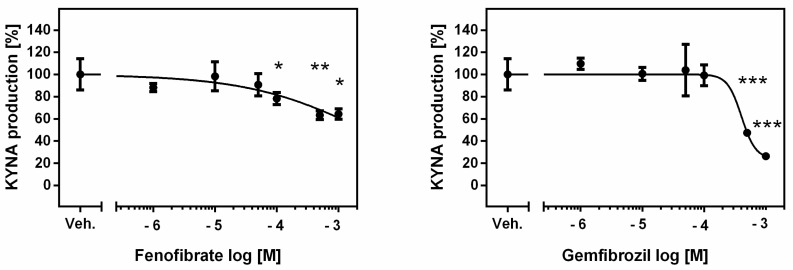
Effect of fibrates on KAT II activity in rat kidneys in vitro. Data presenting the enzymatic activity as percentage of KYNA synthesis, mean ± SD, *n* = 3, are plotted against drug concentration on a logarithmic scale. ANOVA followed by Tukey’s multiple comparison test. The figure was prepared in GraphPad Prism 6. * *p <* 0.05, ** *p <* 0.01, *** *p <* 0.001.

The mean KYNA production by KAT II in the tested kidney homogenates in the presence of 2 μM KYN was 93.71 ± 16.98 pmol/mg tissue. Fenofibrate at 100 µM, 500 µM and 1 mM concentrations lowered the standard KAT II activity in rat kidneys in vitro to 78% (*p* < 0.05), 63% (*p* < 0.01) and 64% (*p* < 0.05), respectively (Figure 3).

Compared to fenofibrate, gemfibrozil at 500 µM and 1 mM concentrations decreased the KAT II activity more efficiently in rat kidneys in vitro to 47% (*p* < 0.001) and 26% (*p* < 0.001) of the control value, respectively.

A summary of the determined fibrate effects on KYNA production and KAT activity in rat kidneys in vitro is presented in Table 2.

## 4. Discussion

In this article, we present the original observation of the decreased KYNA production and inhibition of KAT activity in rat kidneys in vitro by drugs which lower the triglyceride level, i.e., fenofibrate and gemfibrozil. The presented findings suggest a potentially protective effect of this class of drugs on kidney tissue.

Although CKD pathogenesis is multifactorial, the activation of the KYN pathway in the course of disease is gaining increasing scientific interest. The circulating levels of KYN, KYNA and other kynurenines are related to the severity of diabetic CKD [30,31]. Similarly, the correlation between plasma KYNA levels, immune system activation and CKD severity was described in animal models and in patients with autosomal dominant polycystic kidney disease (ADPKD) [32,33].

Chronic inflammation, which is directly involved in kidney damage, may also affect the course of the disease indirectly through the KYN pathway. KYN and KYNA as ligands of AHR may contribute to oxidative stress and mitochondrial dysfunction [11,13]. An excessive activation of AHR leads to apoptosis and specific kidney damage, i.e., podocyte damage, glomerular injury and interstitial fibrosis [34]. Rats fed a high-Trp diet developed focal tubule interstitial injuries, had higher serum levels of KYN metabolites and showed higher serum and kidney AHR expression compared to controls [35]. Since KYN metabolites are defined as uremic toxins, fibrate-evoked inhibition of KYNA synthesis should be considered as a novel mechanism of kidney protection associated with a decreased formation of the AHR ligand. 

Hypertriglyceridemia is the most common lipid abnormality in patients with impaired kidney functions, especially with advanced CKD [36]. Inappropriate lipid levels are correlated with a high cardiovascular risk and directly predispose patients to CKD development [37]. Hypertriglyceridemia is also significantly related to a worse prognosis of immunoglobulin A (IgA) nephropathy, commonly leading to CKD [38]. Thus, lipid abnormality management in patients with kidney damage seems vital, not only in terms of cardiovascular prevention and mortality risk reduction, but especially as a way to stabilize kidney function and to delay the need for kidney replacement therapies. 

Experimental and clinical studies support the hypothesis that fibrates may serve as nephroprotective agents and that their possible beneficial effect to kidney functions may go beyond the standard lipid-lowering effect. The pleiotropic effects of fibrates include decreased kidney damage by mitochondrial dysfunction improvements, as shown in an ADPKD model [39]. Fibrates reduced the oxidative stress level in a nicotine-induced model of AKI [40], in an ischemia reperfusion AKI model [41] and in a kidney fibrosis model [42]. Clofibrate decreased AHR protein levels in Wistar rats, suggesting metabolic improvement through AHR signaling inhibition [43]. Gemfibrozil was shown to lower glomerular damage, decrease proteinuria and improve the kidney function in various animal models [44,45,46]. Correspondingly, in a large cohort of CKD patients, a significantly lower cardiovascular risk and a decreased need for dialysis in patients receiving gemfibrozil were found [47]. Only some data raise the question about the usefulness of triglyceride-lowering drugs in kidney function preservation [48] and cardiovascular prevention in patients with preexisting kidney function declines [49]. 

Our study seems to support the role of fibrates in cardiovascular prevention in the CKD population by decreasing KYNA synthesis in the kidneys. Gemfibrozil showed a stronger inhibition of KYNA production in comparison to fenofibrate. As the inhibition of KYNA formation in the kidneys can be considered nephroprotective, gemfibrozil should appear as more effective in kidney function preservation. Indeed, in comparison with fenofibrate, a therapeutically equivalent dose of gemfibrozil led to significantly higher urine output in mice [50]. 

Nevertheless, it cannot be excluded that a decline in KYNA levels may actually exert negative effects on the kidney morphology, as some studies have shown that KYNA may prevent kidney damage in rats after heatstroke [51] and counteract kidney injury in ischemia reperfusion-induced AKI animal models [52].

In the context of the available literature, the observed decrease in kidney KYNA production may rather serve as a prognostic marker of fibrate effectiveness. Renin-angiotensin system inhibitors reduce kidney KYNA synthesis [53,54]. Furthermore, lowered serum levels of the KYNA precursor KYN in diabetic CKD patients receiving renin-angiotensin system inhibitors, compared to nonusers, were demonstrated [55]. Conversely, a higher KYN/Trp ratio in diabetic CKD patients was significantly correlated with albuminuria and the predicted responsiveness to angiotensin II type 1 receptor blockers [56]. Thus, we suggest that inhibition of KYNA formation by fibrates can serve as a diagnostic tool for the evaluation of drug effectiveness and for patient outcome predictions, especially in terms of a kidney function decline. 

Our study seem to have potential clinical value, especially considering that the effect observed here occurred at concentrations (~100 µM) which are in the range of serum fibrate levels found in healthy subjects (30–50 µM) [57], and is comparable with other in vitro studies utilizing even higher fibrate concentrations [58]. 

Nevertheless, there are some limitations to our study. To explore whether fenofibrate and gemfibrozil affect KYNA synthesis and KAT activity in rat kidneys, the experiments were performed under in vitro conditions on male Wistar rats, widely used in animal research. Further research using CKD models and measurements of kidney functions is required to assess the effectiveness of the tested fibrates under in vivo conditions. Furthermore, appropriate clinical studies should follow. 

## 5. Conclusions

In conclusion, the presented study indicates a novel mechanism of action of fibrates. Inhibition of KYNA synthesis and KAT activity in rat kidneys in vitro by the PPAR-α agonists selected in this study suggests their possible involvement in kidney function regulation. A lowered synthesis of uremic toxins by decreasing the KAT activity suggests a nephroprotective effect of fibrates. Future studies are needed to explore the role of fibrates in the synthesis of other KYN pathway compounds in the kidneys and its clinical implications.

## Figures and Tables

**Table 1:** List of reagents used in presented study, according to their appearance in the text.

	Name of Reagent	Catalogue Number	Manufacturer
1.	L-kynurenine sulfate salt	K3750	Sigma-Aldrich (St. Louis, MO, USA)
2.	fenofibrate	F6020	Sigma-Aldrich (St. Louis, MO, USA)
3.	gemfibrozil	G9518	Sigma-Aldrich (St. Louis, MO, USA)
4.	dimethyl sulfoxide (DMSO)	D1435	Sigma-Aldrich (St. Louis, MO, USA)
5.	sodium chloride	S7653	Sigma-Aldrich (St. Louis, MO, USA)
6.	potassium chloride	P9333	Sigma-Aldrich (St. Louis, MO, USA)
7.	magnesium sulfate heptahydrate	M7506	Sigma-Aldrich (St. Louis, MO, USA)
8.	calcium chloride anhydrous	C1016	Sigma-Aldrich (St. Louis, MO, USA)
9.	sodium phosphate dibasic dodecahydrate	04273	Sigma-Aldrich (St. Louis, MO, USA)
10.	sodium phosphate mono-basic dihydrate	1.06345	Sigma-Aldrich (St. Louis, MO, USA)
11.	glucose	G8270	Sigma-Aldrich (St. Louis, MO, USA)
12.	Trizma base	T1503	Sigma-Aldrich (St. Louis, MO, USA)
13.	acetic acid	A6283	Sigma-Aldrich (St. Louis, MO, USA)
14.	pyridoxal 5′-phosphate hydrate	P9255	Sigma-Aldrich (St. Louis, MO, USA)
15.	2-mercaptoethanol	M3148	Sigma-Aldrich (St. Louis, MO, USA)
16.	pyruvate	P2256	Sigma-Aldrich (St. Louis, MO, USA)
17.	glutamine	D9003	Sigma-Aldrich (St. Louis, MO, USA)
18.	water for HPLC	JTB-4218.2500	J.T. Baker Chemicals (Phillipsburg, NJ, USA)
19.	acetonitrile	34851	Sigma-Aldrich (St. Louis, MO, USA)
20.	zinc acetate dehydrate	1088021000	Sigma-Aldrich (St. Louis, MO, USA)
21.	sodium acetate trihydrate	1062671000	Sigma-Aldrich (St. Louis, MO, USA)
22.	acetic acid	A6283	Sigma-Aldrich (St. Louis, MO, USA)

**Table 2:** Summary of fibrate effects on KYNA formation and KAT activity in rat kidneys in vitro. Data shown as a percentage of control KYNA synthesis. Fenofibrate and gemfibrozil concentrations that did not show any effect on KYNA production or KAT activity (0.001 mM, 0.01 mM and 0.05 mM) are not presented. NS, non-significant. * *p <* 0.05; ** *p <* 0.01, *** *p <* 0.001 vs. control (ANOVA).

	Fenofibrate (mM)			Gemfibrozil (mM)		
	0.1 mM	0.5 mM	1 mM	0.1 mM	0.5 mM	1 mM
KYNA production	72% *	60% ***	51% ***	66% ***	58% ***	41% ***
KAT I activity	NS	68% *	59% *	68% *	56% **	52% **
KAT II activity	78% *	63% **	64% *	NS	47% ***	26% ***

## Data Availability

The datasets generated during the current study are available from the corresponding author upon reasonable request.

## References

[1] Mathew R.O., Rosenson R.S., Lyubarova R., Chaudhry R., Costa S.P., Bangalore S., Sidhu M.S. (2021). Concepts and Controversies: Lipid Management in Patients with Chronic Kidney Disease. Cardiovasc. Drugs Ther..

[2] Vanholder R., Massy Z., Argiles A., Spasovski G., Verbeke F., Lameire N. (2005). Chronic Kidney Disease as Cause of Cardiovascular Morbidity and Mortality. Nephrol. Dial. Transplant..

[3] Ferro C.J., Mark P.B., Kanbay M., Sarafidis P., Heine G.H., Rossignol P., Massy Z.A., Mallamaci F., Valdivielso J.M., Malyszko J. (2018). Lipid Management in Patients with Chronic Kidney Disease. Nat. Rev. Nephrol..

[4] Florens N., Calzada C., Lyasko E., Juillard L., Soulage C.O. (2016). Modified Lipids and Lipoproteins in Chronic Kidney Disease: A New Class of Uremic Toxins. Toxins.

[5] Gyebi L., Soltani Z., Reisin E. (2012). Lipid Nephrotoxicity: New Concept for an Old Disease. Curr. Hypertens. Rep..

[6] Kostapanos M.S., Florentin M., Elisaf M.S. (2013). Fenofibrate and the Kidney: An Overview. Eur. J. Clin. Invest..

[7] Zhao X., Li L.-Y. (2008). PPAR-Alpha Agonist Fenofibrate Induces Renal CYP Enzymes and Reduces Blood Pressure and Glomerular Hypertrophy in Zucker Diabetic Fatty Rats. Am. J. Nephrol..

[8] Hakimizadeh E., Tadayon S., Zamanian M.Y., Soltani A., Giménez-Llort L., Hassanipour M., Fatemi I. (2023). Gemfibrozil, a Lipid-lowering Drug, Improves Hepatorenal Damages in a Mouse Model of Aging. Fundam. Clin. Pharmacol..

[9] Davidson M., Rashidi N., Nurgali K., Apostolopoulos V. (2022). The Role of Tryptophan Metabolites in Neuropsychiatric Disorders. Int. J. Mol. Sci..

[10] Schefold J.C., Zeden J.P., Fotopoulou C., Von Haehling S., Pschowski R., Hasper D., Volk H.D., Schuett C., Reinke P. (2009). Increased Indoleamine 2,3-Dioxygenase (IDO) Activity and Elevated Serum Levels of Tryptophan Catabolites in Patients with Chronic Kidney Disease: A Possible Link between Chronic Inflammation and Uraemic Symptoms. Nephrol. Dial. Transplant..

[11] Mor A., Kalaska B., Pawlak D. (2020). Kynurenine Pathway in Chronic Kidney Disease: What’s Old, What’s New, and What’s Next?. Int. J. Tryptophan Res..

[12] Hughes T.D., Güner O.F., Iradukunda E.C., Phillips R.S., Bowen J.P. (2022). The Kynurenine Pathway and Kynurenine 3-Monooxygenase Inhibitors. Molecules.

[13] Di Natale B.C., Murray I.A., Schroeder J.C., Flaveny C.A., Lahoti T.S., Laurenzana E.M., Omiecinski C.J., Perdew G.H. (2010). Kynurenic Acid Is a Potent Endogenous Aryl Hydrocarbon Receptor Ligand That Synergistically Induces Interleukin-6 in the Presence of Inflammatory Signaling. Toxicol. Sci..

[14] Curran C.S., Kopp J.B. (2022). Aryl Hydrocarbon Receptor Mechanisms Affecting Chronic Kidney Disease. Front. Pharmacol..

[15] Yeh Y.-C., Huang M.-F., Liang S.-S., Hwang S.-J., Tsai J.-C., Liu T.-L., Wu P.-H., Yang Y.-H., Kuo K.-C., Kuo M.-C. (2016). Indoxyl Sulfate, Not p-Cresyl Sulfate, Is Associated with Cognitive Impairment in Early-Stage Chronic Kidney Disease. Neurotoxicology.

[16] Lin C.S., Hung S.F., Huang H.S., Ma M.C. (2015). Blockade of the N-Methyl-D-Aspartate Glutamate Receptor Ameliorates Lipopolysaccharide-Induced Renal Insufficiency. PLoS ONE.

[17] Dou L., Sallée M., Cerini C., Poitevin S., Gondouin B., Jourde-Chiche N., Fallague K., Brunet P., Calaf R., Dussol B. (2015). The Cardiovascular Effect of the Uremic Solute Indole-3 Acetic Acid. J. Am. Soc. Nephrol..

[18] Ostapiuk A., Urbanska E.M. (2022). Kynurenic Acid in Neurodegenerative Disorders—Unique Neuroprotection or Double-edged Sword?. CNS Neurosci. Ther..

[19] Bądzyńska B., Zakrocka I., Sadowski J., Turski W.A., Kompanowska-Jezierska E. (2014). Effects of Systemic Administration of Kynurenic Acid and Glycine on Renal Haemodynamics and Excretion in Normotensive and Spontaneously Hypertensive Rats. Eur. J. Pharmacol..

[20] Bądzyńska B., Zakrocka I., Turski W.A., Olszyński K.H., Sadowski J., Kompanowska-Jezierska E. (2020). Kynurenic Acid Selectively Reduces Heart Rate in Spontaneously Hypertensive Rats. Naunyn. Schmiedebergs. Arch. Pharmacol..

[21] Vanholder R., Nigam S.K., Burtey S., Glorieux G. (2022). What If Not All Metabolites from the Uremic Toxin Generating Pathways Are Toxic? A Hypothesis. Toxins.

[22] Chen Y., Zelnick L.R., Wang K., Katz R., Hoofnagle A.N., Becker J.O., Hsu C.-Y., Go A.S., Feldman H.I., Mehta R.C. (2021). Association of Tubular Solute Clearances with the Glomerular Filtration Rate and Complications of Chronic Kidney Disease: The Chronic Renal Insufficiency Cohort Study. Nephrol. Dial. Transplant..

[23] Pawlak K., Myśliwiec M., Pawlak D. (2010). Kynurenine Pathway—A New Link between Endothelial Dysfunction and Carotid Atherosclerosis in Chronic Kidney Disease Patients. Adv. Med. Sci..

[24] Emami F., Hariri A., Matinfar M., Nematbakhsh M. (2020). Fenofibrate-Induced Renal Dysfunction, Yes or No?. J. Res. Med. Sci..

[25] Zarnowski T., Tulidowicz-Bielak M., Zarnowska I., Mitosek-Szewczyk K., Wnorowski A., Jozwiak K., Gasior M., Turski W.A. (2017). Kynurenic Acid and Neuroprotective Activity of the Ketogenic Diet in the Eye. Curr. Med. Chem..

[26] Żarnowska I., Wróbel-Dudzińska D., Tulidowicz-Bielak M., Kocki T., Mitosek-Szewczyk K., Gasior M., Turski W.A. (2019). Changes in Tryptophan and Kynurenine Pathway Metabolites in the Blood of Children Treated with Ketogenic Diet for Refractory Epilepsy. Seizure.

[27] Clause B.T. (1993). The Wistar Rat as a Right Choice: Establishing Mammalian Standards and the Ideal of a Standardized Mammal. J. Hist. Biol..

[28] Rocha N.N. (2022). Are Wistar Rats the Most Suitable Normotensive Controls for Spontaneously Hypertensive Rats to Assess Blood Pressure and Cardiac Structure and Function?. Int. J. Cardiovasc. Sci..

[29] Gramsbergen J.B.P., Schmidt W., Turski W.A., Schwarcz R. (1992). Age-Related Changes in Kynurenic Acid Production in Rat Brain. Brain Res..

[30] Zakrocka I., Załuska W. (2022). Kynurenine Pathway in Kidney Diseases. Pharmacol. Reports.

[31] Debnath S., Velagapudi C., Redus L., Thameem F., Kasinath B., Hura C.E., Lorenzo C., Abboud H.E., O’Connor J.C. (2017). Tryptophan Metabolism in Patients with Chronic Kidney Disease Secondary to Type 2 Diabetes: Relationship to Inflammatory Markers. Int. J. Tryptophan Res..

[32] Klawitter J., Jackson M.J., Smith P.H., Hopp K., Chonchol M., Gitomer B.Y., Cadnapaphornchai M.A., Christians U., Klawitter J. (2022). Kynurenines in Polycystic Kidney Disease. J. Nephrol..

[33] Pires A.S., Gupta S., Barton S.A., Vander Wall R., Tan V., Heng B., Phillips J.K., Guillemin G.J. (2022). Temporal Profile of Kynurenine Pathway Metabolites in a Rodent Model of Autosomal Recessive Polycystic Kidney Disease. Int. J. Tryptophan Res..

[34] Mo Y., Lu Z., Wang L., Ji C., Zou C., Liu X. (2020). The Aryl Hydrocarbon Receptor in Chronic Kidney Disease: Friend or Foe?. Front. Cell Dev. Biol..

[35] Hu D., Liu J., Yu W., Li C., Huang L., Mao W., Lu Z. (2023). Tryptophan Intake, Not Always the More the Better. Front. Nutr..

[36] Cader A., Stępniewska J., Różański J. (2022). Lipid Disorders—The Comparison between General Population and Haemodialyzed Patients. Will the Oral Fat Tolerance Test Improve the Diagnostic?. Acta Biochim. Pol..

[37] Rizk J.G., Hsiung J.-T., Arif Y., Hashemi L., Sumida K., Kovesdy C.P., Kalantar-Zadeh K., Streja E. (2023). Triglycerides and Renal Outcomes According to Albuminuria and in Consideration of Other Metabolic Syndrome Components in Diabetic US Veterans. Am. J. Nephrol..

[38] Liu S., Lu Z., Fu Z., Li H., Gui C., Deng Y. (2023). Clinicopathological Characteristics and Outcomes of Immunoglobulin A Nephropathy with Different Types of Dyslipidemia: A Retrospective Single-Center Study. Kidney Blood Press. Res..

[39] Liu X., Du H., Sun Y., Shao L. (2022). Role of Abnormal Energy Metabolism in the Progression of Chronic Kidney Disease and Drug Intervention. Ren. Fail..

[40] Chakkarwar V., Kawtikwar P. (2021). Fenofibrate Prevents Nicotine-Induced Acute Kidney Injury: Possible Involvement of Endothelial Nitric Oxide Synthase. Indian J. Nephrol..

[41] Kaur J., Kaur T., Sharma A.K., Kaur J., Yadav H.N., Pathak D., Singh A.P. (2021). Fenofibrate Attenuates Ischemia Reperfusion-induced Acute Kidney Injury and Associated Liver Dysfunction in Rats. Drug Dev. Res..

[42] Xu S.-S., Li S., Zhang X.-X., Qi J.-Z., Li G.-Y. (2020). A Study on the Role and Mechanism of Fenofibrate in Mice Renal Fibrosis Induced by Unilateral Ureteral Obstruction. Sichuan Da Xue Xue Bao. Yi Xue Ban.

[43] Shaban Z., El-Shazly S., Ishizuka M., Kimura K., Kazusaka A., Fujita S. (2004). PPAR?-Dependent Modulation of Hepatic CYP1A by Clofibric Acid in Rats. Arch. Toxicol..

[44] Shields C.A., Poudel B., McPherson K.C., Brown A.K., Ekperikpe U.S., Browning E., Sutton L., Cornelius D.C., Williams J.M. (2020). Treatment With Gemfibrozil Prevents the Progression of Chronic Kidney Disease in Obese Dahl Salt-Sensitive Rats. Front. Physiol..

[45] Hosseinzadeh A., Goudarzi M., Fatemi I., Khodayar M.J., Mehrzadi S., Khalili H.R., Karimi M.A. (2020). Gemfibrozil Attenuates Doxorubicin Induced Toxicity in Renal Tissues of Male Rats by Reducing the Oxidative Insult and Inflammation. Biotech. Histochem..

[46] Miglio G., Rosa A.C., Rattazzi L., Grange C., Camussi G., Fantozzi R. (2012). Protective Effects of Peroxisome Proliferator-Activated Receptor Agonists on Human Podocytes: Proposed Mechanisms of Action. Br. J. Pharmacol..

[47] Yen C.-L., Fan P.-C., Lin M.-S., Lee C.-C., Tu K.-H., Chen C.-Y., Hsiao C.-C., Hsu H.-H., Tian Y.-C., Chang C.-H. (2021). Fenofibrate Delays the Need for Dialysis and Reduces Cardiovascular Risk Among Patients With Advanced CKD. J. Clin. Endocrinol. Metab..

[48] Khurana N., James S., Coughlan M.T., MacIsaac R.J., Ekinci E.I. (2022). Novel Therapies for Kidney Disease in People With Diabetes. J. Clin. Endocrinol. Metab..

[49] Ananthakrishnan S., Kaysen G.A. (2016). Treatment of Hyperlipidemia Changes With Level of Kidney Function—Rationale. Adv. Chronic Kidney Dis..

[50] Song C., Clark S.M., Vaughn C.N., Nicholson J.D., Murphy K.J., Mou T.C.M., Schwarcz R., Hoffman G.E., Tonelli L.H. (2018). Quantitative Analysis of Kynurenine Aminotransferase II in the Adult Rat Brain Reveals High Expression in Proliferative Zones and Corpus Callosum. Neuroscience.

[51] Hsieh Y.C., Chen R.F., Yeh Y.S., Lin M.T., Hsieh J.H., Chen S.H. (2011). Kynurenic Acid Attenuates Multiorgan Dysfunction in Rats after Heatstroke. Acta Pharmacol. Sin..

[52] Arora S., Kaur T., Kaur A., Singh A.P. (2014). Glycine Aggravates Ischemia Reperfusion-Induced Acute Kidney Injury through N-Methyl-D-Aspartate Receptor Activation in Rats. Mol. Cell. Biochem..

[53] Zakrocka I., Targowska-Duda K.M., Wnorowski A., Kocki T., Jóźwiak K., Turski W.A. (2019). Angiotensin II Type 1 Receptor Blockers Decrease Kynurenic Acid Production in Rat Kidney in Vitro. Naunyn. Schmiedebergs. Arch. Pharmacol..

[54] Zakrocka I., Kocki T., Turski W.A. (2017). The Effect of Three Angiotensin-Converting Enzyme Inhibitors on Kynurenic Acid Production in Rat Kidney in Vitro. Pharmacol. Rep..

[55] Cernaro V., Loddo S., Macaione V., Ferlazzo V.T., Cigala R.M., Crea F., De Stefano C., Genovese A.R.R., Gembillo G., Bolignano D. (2020). RAS Inhibition Modulates Kynurenine Levels in a CKD Population with and without Type 2 Diabetes Mellitus. Int. Urol. Nephrol..

[56] Wu M.-H., Lin C.-N., Chiu D.T.-Y., Chen S.-T. (2020). Kynurenine/Tryptophan Ratio Predicts Angiotensin Receptor Blocker Responsiveness in Patients with Diabetic Kidney Disease. Diagnostics.

[57] Balfour J.A., McTavish D., Heel R.C. (1990). Fenofibrate. Drugs.

[58] Liu A., Yang J., Huang X., Xiong J., Wong A.H.-H., Chang L., Dai R. (2012). Relaxation of Rat Thoracic Aorta by Fibrate Drugs Correlates with Their Potency to Disturb Intracellular Calcium of VSMCs. Vascul. Pharmacol..

